# Current Status and Prospects of Pine Wilt Disease Management with Phytochemicals—A Review

**DOI:** 10.3390/plants13152129

**Published:** 2024-08-01

**Authors:** Quanhong Zhang, Guiling Si, Liusheng Chen, Lili Hu, Gaofeng Cui, Min Wang, Danyang Zhao

**Affiliations:** 1 College of Plant Protection, South China Agricultural University, Guangzhou 510642, China; hqz5321@163.com (Q.Z.); guiling2020163@163.com (G.S.); 2 Guangdong Provincial Key Laboratory of Silviculture, Protection and Utilization, Guangdong Academy of Forestry, Guangzhou 510520, China; lshchen2008@163.com (L.C.); hulili0113@163.com (L.H.)

**Keywords:** pine wilt disease, phytochemicals, research and application, nematicidal activity, current status and prospects

## Abstract

PWD (pine wilt disease) is a devastating forest disease caused by the *Bursaphelenchus xylophilus*, which is the major invasive species in Asian and European countries. To control this disease, fumigation, pesticide injection, and clear cutting of epidemic trees have been widely used. But these management strategies have many limitations in terms of the effectiveness and environmental impacts, especially for the overuse of chemical pesticides. Thus, PCs (phytochemicals), the various compounds extracted from plants, have drawn extensive attention owing to their special characteristics, including abundant sources, low toxicity, high efficacy, and easy degradation. This review provides an overview of the current status of using PCs as alternative approaches to manage PWD. It discusses the efficacy of various PCs, the factors influencing their nematicidal activity, and their mechanism of action against *B. xylophilus*. These results will reveal the application of PCs in combating these devastating diseases and the necessity for further research.

## 1. Introduction

PWD (pine wilt disease) is a devastating forest disease, resulting in forest ecosystem destruction, environmental disruption, loss of biodiversity, and economic losses [1,2,3]. It is caused by the PWN (pine wood nematode), *Bursaphelenchus xylophilus* (Tylenchida, Aphelenchoididae), a migratory endoparasite. *B*. *xylophilus* is native to North America and was introduced into Japan at the start of the 20th century [4]. It then spread to other countries in Asia, such as China and South Korea. The PWN was also introduced into Portugal and Spain due to the global trade, and it could spread to more areas without global quarantine efforts. PWD is characterized by multiple transmission pathways, concealed infection sites, rapid disease progression, and long incubation periods [5]. PWD is mainly transmitted by artificially (over long distances) and insect vectors (over short distances). The insect vectors are primarily Cerambycidae insects, as the dispersal type fourth-stage nematodes could enter the trachea of longicorn beetles within the pine tree. When the longicorn beetles move to healthy trees for feeding and oviposition, the PWN escape and transfer to new hosts under increased carbon dioxide concentration in the trachea [6]. Currently, the PWN can naturally infect 47 pine tree and 14 non-pine tree species [7]. Once a tree is infected, the PWN move through the resin canals, feed in the xylem and phloem, cause obstruction in the water conveyance system of trees, and lead to wilting or death of most infected trees within approximately six months [4]. *Pinus* species are important timber resources that can be used in many industries, such as architecture, furniture manufacturing, papermaking, and woodworking. They are also crucial components in forest ecosystems, which play significant roles in maintaining ecological balance and protecting soil and water sources. Therefore, PWD poses a huge threat to global forest ecosystems and timber trades.

Prevention and control of PWD mainly rely on disease quarantine, epidemiological surveillance, removal of infected trees, control of vector beetles, and injection of nematicides into pine trees [7,8,9,10,11]. Chemical control is an important manner of PWN management, since it offers rapid and effective control, broad-spectrum nematicidal activity, and strong controllability. However, chemical control also has certain drawbacks. For instance, the effectiveness of application tools is limited in complex forest terrain with dense undergrowth, and labor costs are high. The hidden nature of the PWN within the host tree not only makes it difficult to detect early infections, but also hinders the effectiveness of chemical control. Drones are used for nematicides application, it is susceptible to weather conditions and can cause drift contamination. Nematicides are resistant to adhering to pine needles, resulting in low absorption rates. Furthermore, long-term use of chemical pesticides may lead to the development of resistance and adverse effects in the ecological environment [12,13,14,15]. Therefore, more efficient, environmentally friendly, and sustainable pesticides are needed for the prevention and control of PWD.

PCs (phytochemicals) are chemical compounds found in plants, which play crucial roles in growth and development, and defense against pests and diseases. They encompass alkaloids, polysaccharides, volatile oils, terpenes, polyphenols, etc. PCs have wide-ranging applications in agriculture, medicine, healthcare, and food industries. Extensive research has revealed that numerous PCs and their analogues possess the ability to effectively eliminate nematodes, akin to chemical nematicides [16,17,18,19,20,21]. Moreover, PCs have the advantages of low toxicity and low risk for environmental pollution. They can effectively kill pests and diseases, suppress their spread, and enhance the plant immune system [22,23]. PCs have good biodegradability and do not persist in the environment, reducing pollution to soil and water [16,24]. Furthermore, PCs can be obtained through plant extraction or synthesis, making them sustainable and renewable. Therefore, PCs are suggested as alternatives and/or supplements for chemical pesticides, and they may have great potential and prospect in PWD management.

This review systematically summarizes and analyzes the current research and applications of PCs in the control of PWD. Various kinds of PCs are collected, such as plant extracts, plant essential oils, plant secondary metabolites, and derivatives. Many aspects are involved, including the examination of effects, influence factors, and mechanisms of action. These results will enhance our understanding of the current status of PCs in controlling PWD, and provide a basis for further research and application of PCs.

## 2. Research Analysis of Phytochemicals on Pine Wilt Disease Control

**Figure 1:**
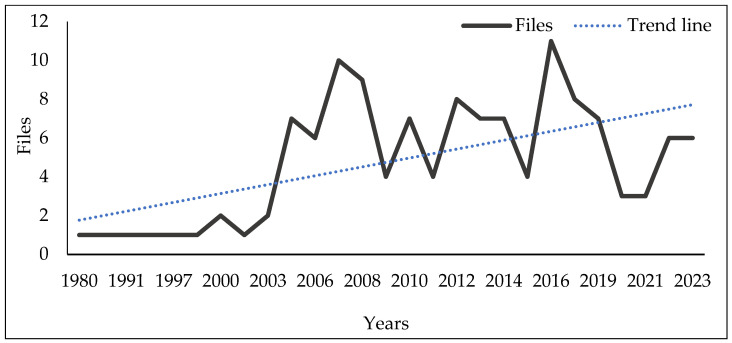
Published literatures on applying phytochemicals for pine wilt disease management. Documents were searched using the keywords, “*Bursaphelenchus xylophilus*” and “Pine Wilt Disease”, in CNKI and WOS, following by manual screening with phytochemical-related words (through 1 January 2024).

Since *B. xylophilus* was identified in 1971 as the pathogenic organism responsible for PWD [25], researchers began to study the physiological characteristics, pathogenic mechanisms, and control methods of the PWN. Here, research analysis is carried out at first. The literature sources were retrieved from the WOS (Web of Science; https://www.webofscience.com; accessed on 31 December 2023) and the CNKI (China National Knowledge Infrastructure; http://www.cnki.net/; accessed on 31 December 2023) using the keywords “*Bursaphelenchus xylophilus*” and “Pine Wilt Disease”, followed by manual screening with phytochemical-related words. The usage of PCs for PWD management can be traced back to 1980, and a total of 128 books, papers, theses, and conference reports were recorded in Chinese and English (Table S1). The temporal span of these records ranged from 1980 to 2023 (Figure 1) and they showed an overall trend of increase despite occasional decreases in certain years, which indicates increasing attention on these topics.

**Figure 2:**
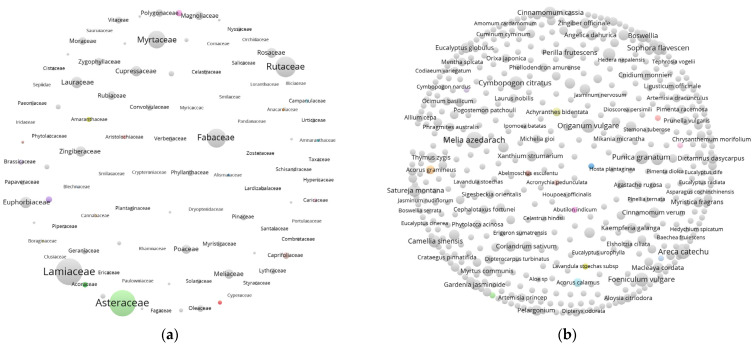
Network visualization of different plant species with nematicidal activity against *Bursaphelenchus xylophilus* among 138 families (**a**) and 538 species (**b**). The statistical and categorization analysis of all plants mentioned in the retrieve literatures, allowing for duplicate species, and compiled into a text format for network visualization analysis using VOSviewer 1.6.19.

According to the literature data obtained (as of 2023), 749 plant species from 171 families have been studied for their potential in controlling the PWN; 540 species from 138 families have shown nematicidal activity, with 555 active substances identified (Table S2). Lamiaceae is the most extensively studied family, followed by Asteraceae, Rutaceae, Fabaceae, and Myrtaceae. Only a few species are mentioned multiple times, such as *Areca catechu*, *Cymbopogon citratus*, *Foeniculum vulgare*, *Melia azedarach*, *Punica granatum*, and *Sophora flavescens* (Figure 2). The scientific names of some plants have been corrected due to their changed classification status. Additionally, 91 different combinations of compounds and 433 derivatives have been synthesized through chemical processes. The derivative substrates mainly include CPT (camptothecin), podophyllotoxin, matrine, coumarin, and 3-acylbarbituric acid. It is important to notice that 87 plant species, 150 substances, and 62 plant extracts have shown high-potential applications. The total number of plants and substances used in the control of the PWN is accumulating, but the newly reported compounds are decreasing, which are shifting toward more specialized and detailed descriptions of the substances. In early research, most studies focused on screening the nematicidal activity of crude extracts from various plants. With the development of technology, chromatographic analysis, mass spectrometry, and nuclear magnetic resonance have been utilized to identify the specific effective nematicidal components of various crude extracts, and to analyze their composition, structure–activity relationships, and mechanisms of action.

## 3. Analysis Process of Using Phytochemicals to Control *Bursaphelenchus xylophilus*

**Figure 3:**
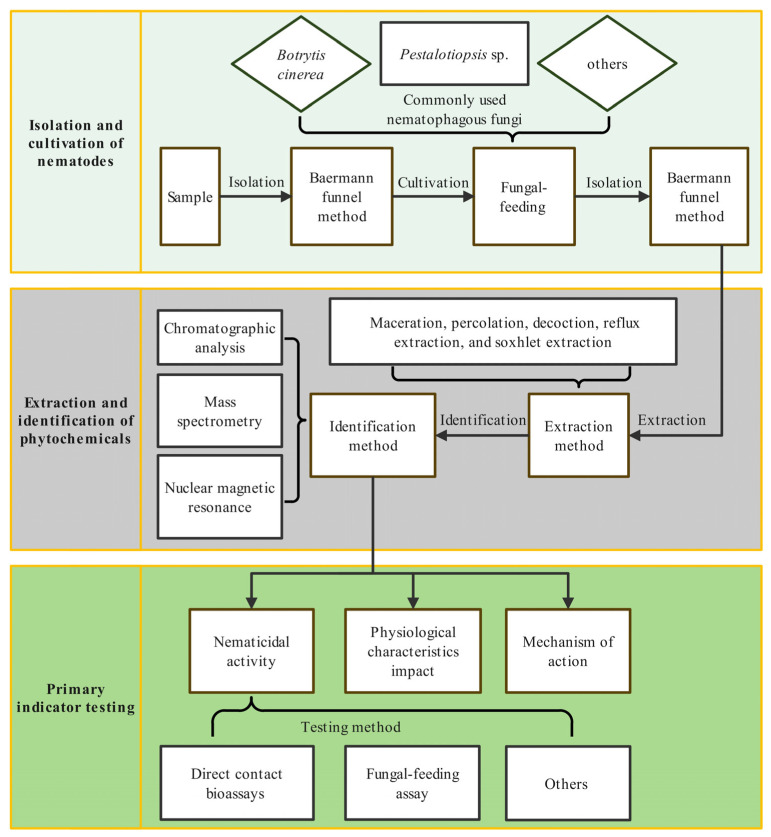
The general research process used in applying phytochemicals to control *Bursaphelenchus xylophilus*. The diagram was created using the software Edraw Max 13.0.5.

Based on the collected documents, a general process of applying PCs to control the PWN is proposed (Figure 3), which includes isolation and cultivation of nematode, extraction and identification of PCs, nematicidal activity and mechanism analysis.

To isolate nematodes, the Baermann funnel method or its advanced method is typically employed, which is based on the water affinity of nematodes. When the nematodes encounter water, they will freely move out from plant tissues and sink due to gravity into the latex tubing connected to the bottom of the funnel, achieving nematode separation. The separated PWNs are usually cultured using *Botrytis cinerea* (76.56%) and *Pestalotiopsis* sp. (17.97%) (Table S3), and it has been demonstrated that the PWN exhibit extremely high fecundity on *B. cinerea* culture medium [26].

The method of extraction commonly used for obtaining the PC involves maceration, percolation, decoction, reflux extraction, and soxhlet extraction. Ethanol is the most frequently used extraction solvent, followed by distilled water and methanol. Furthermore, chromatographic analysis, mass spectrometry, and nuclear magnetic resonance are commonly used to identify the active ingredients in the extracted compounds. As for the nematicidal activity, direct contact bioassay is the most commonly used method indoors, accounting for almost 92.19%. The nematodes are directly exposed to different treatments and most tests involve direct contact soaking, while a smaller number involve soaking of wooden stakes. The nematicidal evaluation using a fungal-feeding assay accounts for 7.03% of total activity assay, which is accompanied by the observation of growth and reproduction. Other methods (0.78%) include spray or injected nematicides (Table S4). The application and efficacy assessment of PCs against the PWN are predominantly conducted indoors, and only a few in the field. The testing indicators were relatively simple before 2012, mainly focusing on the extraction isolation, substance identification, and indoor/outdoor insecticidal activity testing. After 2012, researchers began to focus on the effects of PCs on the growth and development of the PWN, as well as the synergistic effects and mechanisms of action.

## 4. Phytochemicals with High Nematicidal Activity against *Bursaphelenchus xylophilus*

As mentioned above, 87 plant species, 150 substances, and 62 plant extracts have shown significant nematicidal activity against PWD (Table 1). These PCs were classified into alkaloids, terpenes, phenylpropanoids, coumarins, flavonoids, polyphenols, glycosides, sulfurs, polyacetylenes, and related derivatives, based on their chemical structures and properties.

### 4.1. Alkaloids

Alkaloids are alkaline nitrogen compounds commonly found in living organisms and most have complex nitrogen-containing heterocycles [27]. Some nitrogen compounds from plants, which are not alkaline but show significant biological activity, are also classified as alkaloids. Some alkaloids exhibit antitumor, anti-inflammatory, antiviral, antiplatelet aggregation, antiarrhythmic, and antihypertensive properties [28]. *Sophora alopecuroides*, a traditional Chinese medicine, is primarily found in desert and semidesert areas. Over 20 alkaloids had been identified abroad, while 11 alkaloids had been isolated from domestic *S. alopecuroides* in China [29,30,31,32]. Among them, aloperine, a quinolizidine alkaloid, exhibits good nematicidal activity against the PWN. Owing to the widely available material, cost-effective extraction, and developed separation technology, aloperine could be beneficial for the prevention and control of PWD. A bioassay using *B. cinerea* mycelium cultured in an aloperine-containing medium was conducted. The LC_50_/5 d was 0.263 μg/mL, and the nematode mortality rate increased to 94.3% after 15 d. However, the aloperine content in the medium did not significantly decrease after application, suggesting its potential long-lasting effects [33]. Furthermore, aloperine was confirmed to be safe for humans and animals after injecting into pine trees [34]. Aloperine was also injected into the infected black pines with a trunk diameter of 35 cm at Zhongshan Mausoleum in Nanjing. There were no signs of disease and vigorous growth was exhibited after 1 year, while the mortality rate reached 30% in the control area [35]. Later tests showed that 100 μg/mL aloperine resulted in a nematode mortality rate of 62.6% for 7 d in a fungal-feeding assay [36]. As for the structure–activity relationship, aloperine with a secondary amine type nitrogen atom was confirmed to exhibit stronger nematicidal activity than Δ^11^-dehydroaloperine with tertiary amine-type nitrogen atom in 1988, which supported the hypothesis on the structure–activity relationship [37].

CPT, a cytotoxic quinoline alkaloid that inhibits DNA topoisomerase I, was discovered as an anticancer drug in 1966. It exhibits promising potential in the control of PWD through the synthesis of various derivatives or combination with nanomaterials. For example, 7-CH_2_C_6_H_5_-CPT, 7-CHO-CPT, 7-CH_2_OC-OC_6_H_5_-CPT, 10-CH_2_OCOC_6_H_5_-CPT, 20-CH_2_OCOC_6_H_5_-CPT, and 20-F-CPT were screened from 13 synthesized 7-C-substituted 20-(S)-camptothecin derivatives, with LC_50_/24 h values of 2.28, 2.21, 1.37, 1.68, 0.13, and 1.17 mg/L, respectively, which were significantly higher than that of CPT (12.18 mg/L). Although changes in chain length did not significantly affect the activity, it varied greatly with different substituents, possibly owing to the alterations in water solubility and different functional groups [38]. 7-(1-(4-methoxybenzoyl) piperazin-4-yl)-methyl-camptothecin and 7-(1-(2-methoxybenzoyl) piperazin-4-yl)-methyl-camptothecin also achieved LC_50_/24 h values of 6.34 and 6.53 mg/L, respectively, against PWNs. However, there is no regular correlation between the nematicidal activity and the type or position of halogen-containing groups introduced on the benzene ring [39]. A CNDS (CPT nanodrug-loading system) was prepared using an emulsification cross-linking method. This CNDS, with an average particle size of 900 nm, significantly improved the water solubility of CPT. The use of tannic acid for surface modification of the CNDS improved the stability of CPT and increased its adhesion to plant leaves. The nematicidal activity was determined using the immersion method, and the tannic acid/CNDS (98.33%) exhibited a higher nematicidal activity than CNDS (90.48%) [40].

Harmaline, with the chemical formula C_11_H_12_N_2_O, is a β-carboline alkaloid found in the seeds of *Peganum harmala* [41,42]; harmaline exhibited bioactivity with an LC_50_/48 h value of 135.74 µg/mL for mixed-age PWNs [43]. Nineteen harmaline camel-base hydrazide derivatives were synthesized and their nematicidal activity results suggested that N-2,4,6-trimethoxybenzyl-β-carboline-3-carbohydrazide had the strongest toxicity (LC_50_/48 h = 42.49 μg/mL). Harmaline derivatives could delay the development of PWD as treated pine branches continued to secrete resin, while control branches showed significantly reduced or even stopped resin secretion [44]. Furthermore, the nematicidal activity and mechanism of harmaline derivatives were studied. The LC_50_/72 h values against the PWN for the synthesizing compounds F3, F9, F14, and F25, were 30.600, 130.697, 108.660, and 129.318 mg/L, respectively. Compound F14 was found to have a certain inhibitory effect on PWN ACE (acetylcholinesterase), α-carboxylesterase, β-carboxylesterase, and CAT (catalase) [45]. Furthermore, harmine quaternary ammonium derivatives 10, 11, 12, and 13 displayed promising nematicidal effects with LC_50_/48 h values of 1.63, 1.63, 1.75, and 1.44 μg/mL, respectively, and remarkable inhibition effects on ACE, whose IC_50_ values were 0.92, 0.90, 0.82, 0.07 μg/mL in vitro and 17.16, 14.56, 13.63, 3.06 μg/mL in vivo, respectively [46].

Piperine at a concentration of 50 µg/mL could effectively kill PWN juveniles within 6 h, causing rapid curling, aggregation, and death. It also negatively impacted the reproductive and movement abilities of the nematodes. Studies revealed that piperine interacts with the Gln^219^ of the GluCl receptor, which is associated with receptor activation [47]. Drupacine exhibited strong nematotoxicity (ED_50_/72 h = 27.1 µg/mL), which demonstrated suppression of nematode hatch, activity of various life stages, and population numbers [48]. 5′-Methoxywaltherione A and waltherione A were isolated from the roots of *Waltheria indica*, with EC_50_/72 h values against the PWN of 2.13 and 3.54 μg/mL, respectively [49]. The methanol extracts from different parts of *Clausena lansium* were evaluated for their bioactivity against the PWN; the petroleum ether fraction showed the highest activity after extraction with different solvents. Nuclear magnetic resonance analysis revealed the presence of lansiumamide B, and the LC_50_ values of lansiumamide B against the PWN at 24, 48, and 72 h were 8.38, 6.36, and 5.38 mg/L, respectively [50]. Quinoline alkaloids, pyrrolidine alkaloids, and matrine and its derivatives also exhibit good nematicidal activity [51,52,53]. Additionally, the survival rate of the PWN decreased gradually as the matrine concentration increased, showing a significant decrease in the survival rate of PWNs treated with RNA interference (*BxCYP33C9*-/*BxCYP33C4*-/*BxCYP33D3*-/*BxGST3*-/*BxGST1*-/*BxPGP23*-dsRNA) under matrine stress when compared to the control group [54].

### 4.2. Terpenes

Terpenes, also referred to as terpenoids or isoprenoids, are widely found in animal and plant species, originating from the linkage of C5 isoprene units in a head-to-tail arrangement through the intermediary mevalonic acid [55]. Twenty-six PCs were screened for their efficacy in killing PWNs using 96-well cell culture plates, and eight terpenes were selected. Carvacrol, menthol, thymol, citronellol, geraniol, citral, citronellal, and nerol exhibited LC_50_/4 h values of 0.125, 0.985, 0.119, 0.245, 0.540, 0.187, 0.321, 0.865 mg/mL on males (M), and 0.097, 0.894, 0.110, 0.235, 0.415, 0.139, 0.298, 0.926 mg/mL on females (F), respectively [56]. The toxic and propagation effects on PWNs of 28 *Thymus vulgaris* red oil and white oil compounds were examined using direct contact bioassay. Geraniol (LC_50_/24 h = 0.47 mg/mL) was the most toxic compound, followed by thymol (1.08 mg/mL) and carvacrol (1.23 mg/mL) [57].

The nematicidal activity of *Trachyspermum ammi*, *Pimenta dioica*, and *Litsea cubeba* essential oils was tested. Citral and neral were identified using GC-MS (gas chromatography–mass spectrometry), and exhibited strong activity with LC_50_/24 h values of 0.120 and 0.525 mg/mL. It also suggested that the functional group at the C1 position of the benzene ring was crucial for the nematicidal activity [58]. Leaves of *Michelia gioi* were found to exhibit nematicidal activity in screening experiments, particularly its ethyl acetate extract. Parthenolide was identified and a 70.4% mortality rate against PWNs was demonstrated after 48 h treatment at a concentration of 200 μg/mL [59]. Fraxinellone, evodin, and obacunone also exhibited good nematicidal activity, which were isolated from the bark of *Dictamnus dasycarpus*, with LC_50_ values of 9.78, 17.91, and 15.99 μg/mL, respectively. The adjusted mortality rate of fraxinellone could reach over 80% at 20 μg/mL [60]. Two diterpenes, specifically 3-O-(2″,3″-Dimethylbutanoyl)-13-O-dodecanoylingenol and 3-O-(2″, 3″-Dimethylbutanoyl)-13-O-decanoylingenol isolated from *Euphorbia kansui*, were found to demonstrate notable antinematodal activity against the PWN. Both compounds exhibited potent effectiveness at a minimum effective dosage of 5 μg when applied to a cotton ball and continued to exhibit antinematodal properties at 2.5 μg [61]. Ethanolic extracts from 30 plant species were tested for nematicidal activity against the PWN and *Panagrellus redivivus*. *Magnolia grandiflora* leaf extracts showed strong nematicidal effects on both nematodes. A new nematicidal sesquiterpene, 4,5-Epoxy-1(10)E,11(13)-germacradien-12,6-olide, was isolated and characterized from *M. grandiflora* leaves. The compound exhibited significant mortality rates on the nematodes (LC50/72 h = 71 µg/mL). This study marked the first discovery of nematicidal activity in the Magnoliaceae family [62].

### 4.3. Phenylpropanoids

Phenylpropanoids are a common group of aromatic chemical constituents found in traditional Chinese medicinal herbs, with a core structure consisting of one or more C_6_-C_3_ units. Here, “phenylpropanoids” refers to simple phenylpropanoids. Cis-asarone and trans-cinnamyl alcohol have been isolated from the essential oils extracted from *Valeriana jatamansi* and *Liquidambar orientalis*, respectively. Both compounds exhibited strong nematicidal activity against the PWN, with mortality of 100% at 1 mg/mL [63]. Isoeugenol, methyl isoeugenol, eugenol, and methyl eugenol were identified using GC-MS, and exhibited strong activity with LC_50_/24 h values of 0.200, 0.210, 0.480, 0.517 mg/mL, respectively [58]. The 34 compounds associated with the extracts of two cassia oils (LC_50_ = 0.084–0.085 mg/mL) and four cinnamon oils (LC_50_ = 0.064–0.113 mg/mL) showed toxicity toward the PWN. The LC_50_ values of allyl cinnamate, ethyl α-cyanocinnamate, ethyl cinnamate, methyl cinnamate, (E)-cinnamaldehyde, α-methyl-(E)-cinnamaldehyde, (E)-4-methoxycinnamaldehyde, (E)-2-methoxycinnamaldehyde were 0.195, 0.333, 0.114, 0.163, 0.057, 0.131, 0.262, 0.270 mg/mL, respectively. Furthermore, structural characteristics, such as types of functional groups, saturation, and carbon skeleton play vital roles in determining the toxicities to PWNs [64]. The nematicidal constituents of *Cinnamomum verum* oils were identified by GC-MS, and the main compound was cinnamyl acetate (LC_50_/4 h = 32.81 µL/L). Moreover, the nematicidal activity varied with compounds, doses, stages and sex of nematode. [65]. Ultrasonic extraction method was utilized to obtain the methanol extracts of *Kaempferia galanga*; ethyl cinnamate (LC_50_/72 h = 29.7 mg/L) and ethyl *ρ*-methoxyl cinnamate (2.81 mg/L) were identified as the active substances with GC-MS and bioassay analysis [66]. The constituents of essential oils from *Gaultheria fragrantissima* and *Zanthoxylum armatum*, which contain methyl trans-cinnamate and ethyl trans-cinnamate, exhibited nematicidal activity exceeding 90% at 2.0 mg/mL. This study suggested that the nematicidal activity of trans-cinnamate was attributed to the existence of a carbonyl group and a double bond at the α, β-position of carbonyl group [67]. Extracts from 40 medicinal plant species in 27 families were screened for their effectiveness against PWNs. Among them, extracts of *Acorus gramineus*, *Asiasarum sieboldi*, *Illicium verum*, and *K. galanga* exhibited nematicidal activity at a concentration of 2000 µg/mL. Further fractionation of *K. galanga* extracts revealed that ethyl trans-cinnamate and ethyl *ρ*-methoxycinnamate were the active compounds responsible for the observed nematicidal activity, with 100% activity at 60 µg/mL [68]. Research has revealed that extracts from *Zostera marina* exhibit potent nematicidal effects against both the PWN and certain accompanying bacteria. By using a series of extraction and purification techniques, rosmarinic acid was isolated from *Z. marina* extracts. Rosmarinic acid demonstrated significant nematicidal properties, with LC_50_ values of 1.18, 1.05, and 0.95 mg/g at 24, 48, and 72 h, respectively, against the PWN [69].

### 4.4. Coumarins

Coumarins, also known as 1,2-benzopyranone, are chemical compounds in the benzopyrone class. Coumarins are commonly used in food, perfumes, and cosmetics, and some are used in pharmaceuticals and health products owing to their antioxidant and antimicrobial properties [70]. For PWD management, 8-geranyloxypsoralen, imperatorin, and heraclenin were extracted from *Heracleum candicans* following bioassay-guided fractionation, and the LC_50_ values were 188.3, 161.7, and 114.7 mg/L at 72 h, respectively. This might be the initial documentation on nematicidal properties of the Apiaceae [71]. With the application of mass spectrometry and nuclear magnetic resonance data, eight compounds were identified from the ethanol extract of *Stellera chamaejasme* roots. Among them, umbelliferone and daphnoretin were isolated and confirmed to demonstrate noteworthy nematicidal activity against PWNs, with LC_50_ values at 72 h of 3.3 and 65.3 µM, respectively [72]. Bergapten and psoralen, two furocoumarins derived from the leaves of *Ficus carica*, exhibited the nematicidal activity with LC_50_/72 h values of 97.08 and 115.03 μg/mL, respectively, and they could inhibit the activities of amylase, cellulase, and ACE of the PWN [73]. Columbianetin (LC_50_/72 h = 21.83–103.44 µg/mL) and isoimperatorin (17.21–30.91 µg/mL), which were isolated from the ethanol extract of *Hansenia weberbaueriana*, had strong nematicidal activity against the PWN. They were more toxic against the PWN under ultraviolet light and day light circumstance than in darkness [74]. Psoralen, bergapten, and columbianadin had strong nematicidal activity, and the LC_50_/72 h values were 463.32, 430.08, 406.74 μM, respectively [75]. The nematicidal properties of *Angelica pubescens* roots were investigated, osthole and xanthotoxin were discovered, which possess LC_50_ values of 489.17 and 435.66 µM at 72 h, respectively. They were demonstrated to significantly inhibit the ACE of the PWN, and 2H-1-benzopyran-2-one core moiety of coumarins was suggested as the key structure of nematotoxicity [76]. Cindimine, isopimpinellin, marmesin, isoimperatorin, imperatorin, bergapten, osthole, and xanthotoxin from *Cnidium monnieri* fruits and *Angelica dahurica* root also showed strong nematicidal activity against the PWN, with LC_50_ values of 24.73, 92.16, 122.96, 43.08, 35.72, 52.07, 64.93, and 54.68 ug/mL at 72 h, respectively. These eight nematicidal coumarins could inhibit the ACE and Ca^2+^ ATPase of the PWN [77].

### 4.5. Flavonoids

Flavonoids are composed of a single benzene ring attached to a benzo-gamma-pyrone structure, characterized by high chemical stability and reactivity. Flavonoids are widely used as insecticides and fungicides in plant protection [78]. Rotenone is widely present in the root bark of some Fabaceae plants, and it is a highly specific toxic substance with strong contact and stomach poison effects on insects, especially diamondback moth larvae, armyworms, and aphids. The effects of rotenone and six imidazole and oxazole anthelmintic pesticides on PWNs were determined. Rotenone showed much stronger nematicidal activity on the PWN than the ACE-targeting nematicide, phenamiphos, with an LC_50_/120 h value of 1.86 μg/mL [79]. However, rotenone has limited effectiveness and distribution within pine trees due to poor stability and solubility characteristics [80]. Thus, researchers are working on developing alternative formulations for application. For example, a water-based nanosuspension of rotenone had a high encapsulation rate of 81.7 ± 3.5% and a suspension rate of 99.23 ± 0.49%. It also enhanced the stability against photodegradation and an LC_50_/24 h value of 0.38 μg/mL, which was higher than that of rotenone (2.85 μg/mL) [81]. Furthermore, rotenone β-cyclodextrin inclusion complex and HP-β-cyclodextrin inclusion complex were synthesized. The stability under UV light was significantly enhanced and the solubility was increased by 13.3-fold and 233.4-fold, respectively [82]. 

A well-known plant flavonoid, 2-phenyl chromone, had an LD_50_ of 100 μM against the PWN, which could induce embryonic and larval lethality in nematodes [83]. Six nematicidal flavonoids from *S. chamaejasme* were identified, namely ruixianglangdusu B, chamaejasmenin C, 7-methoxyneochamaejasmin A, (+)-chamaejasmine, chamaechromone, and isosikokianin A, with LC_50_/72 h values of 15.7, 2.7, 167.3, 4.7, 36.7, and 2200 μM, respectively [72].

### 4.6. Other Compounds and Extracts

Apart from the categories above, some other PCs have also been applied in the control of the PWN, such as polyphenols, glycosides, aliphatic compounds, sulfides, extracts, etc. 

Polyphenols are a widely distributed group of compounds in plants, with various biological activities such as antioxidant, antibacterial, anti-inflammatory, and anticancer properties [84,85]. Punicalin, corilagin, and punicalagin had strong cytotoxicity with LC_50_/72 h values of 826.96, 868.28, 307.08 μM, respectively. PWNs showed stiffness and the presence of abnormal vesicular structures or cavities in the dead nematode bodies after punicalin treatment. Transcriptome analysis revealed 2575 differential expression unigenes. Many emergency response related pathways and factors changed upon punicalin treatment, including intracellular phagocytosis, phagosome maturation, peroxisome pathways, and the MAPK signaling pathway [75]. Furthermore, according to the enzyme assays in vitro, punicalagin could inhibit the activity of ACE (IC_50_ = 0.60 mM), amylase (0.96 mM), and cellulase (1.24 mM) [86].

Glycosides, a diverse group of plant secondary metabolites, are widely present in plants, consisting of a mainly lipophilic aglycone unit and a hydrophilic glycone unit [87]. The nematicidal properties of *Liriope muscari* roots against the PWN were investigated. Bioassay-guided fractionation yielded four glycosides, namely 1,4-epoxy-cis-eudesm-6-O-β-D-glucopyranoside, 1β,6β-dihydroxy-cis-eudesm-3-ene-6-O-β-D-glucopyranoside, 1α,6β-dihydroxy-5,10-bis-epi-eudesm-4(15)-ene-6-O-β-D- glucopyranoside, and 1α,6β-dihydroxy-cis-eudesm-3-ene-6-O-β-D-glucopyranoside, which exhibited significant nematicidal activity against the PWN with LC_50_ values of 339.76, 82.84, 465.68, and 153.39 μg/mL, respectively [88]. Organosulfur compounds exhibit strong antibacterial activity against both Gram-positive and Gram-negative bacteria [89], and they also possess potent inhibitory effects on the PWN. Dipropyl trisulfide and methyl propyl trisulfide of *Allium cepa* essential oil were coated with 0.5% chitosan to form nanoemulsions. The LC_50_/24 h values against the PWN were 5.01 and 16.60 μg/mL, respectively. Chitosan coating improved the long-term storage stability of nanoemulsions and the persistence of their nematicidal activity [90]. Allyl isothiocyanate showed an LC_50_ of 0.000271%. It could induce changes in nematode morphology and mobility, affect the enzymatic activity and metabolism associated with nematode life processes, and finally lead to the disruption of nematode nervous-system signaling and reduction in detoxification capability [91].

Aliphatic compounds are important organic compounds, which are mainly composed of carbon and hydrogen elements, and may also contain oxygen, nitrogen, sulfur, and other elements. The nematicidal activity of aliphatic compounds against the PWN was evaluated by microwell assays. The LC_50_/48 h values of 3-methylbutyl tiglate, isobutyl 2-methylbutanoate, 3-methylbutyl 2-methylbutanoate, 3-methyl-2-butenyl 2-methylbutanoate, and pentyl 2-methylbutanoate were 0.0218, 0.0284, 0.0326, 0.0402, and 0.0480 mg/mL, respectively [92]. The fumigant activity of trans-2-hexenal against the PWN was observed at LC_10_ (0.162 μL/L) and LC_30_ (0.213 μL/L) dosages after 48 h. Trans-2-hexenal effectively suppressed the dispersal ability of nematodes, and had an impact on nutrient metabolism and digestive enzyme activity [93]. Additionally, the nematicidal activity of aliphatic compounds was evaluated and the relationship between structure and activity was investigated. C_9_-C_11_ alkanols, C_10_-C_11_ 2E-alkenols, C_8_-C_9_ 2E-alkenals, and C_9_-C_10_ alkanoic acids possess over 80% nematicidal activity at 0.125 mg/mL. The nematicidal activity of test compounds varied according to functional groups, chain length, compound, and species [94]. Essential oils from *Coriandrum sativum* showed good nematicidal activity against the PWN, and 26 major compounds were identified. The nematicidal activity of trans-2-decen-1-ol, decanol, trans-2-decenal, octanal, nonanal, decanal, undecanal, and dodecanal were explored, and the nematicidal activity was found to exceed 85% at 1 mg/mL after 24 h [63].

(Z)-ligustilide was identified as the active component in *Angelica tenuissima* root extract at 73.6% of the total content ratio. The LC_50_/24 h value of (Z)-ligustilide against the PWN was 0.24 mg/mL [95]. In addition, α-terthienyl from the *Orixa japonica* exhibited highly potent nematicidal activity against the PWN, with an LC_50_ of 1.95 mg/mL [51]. The ethanol extracts of *H. weberbaueriana* have strong nematicidal activity against the PWN. Two Polyacetylenes, falcarindiol (LC_50_/72 h = 0.95 µg/mL) and falcarinol (7.42 µg/mL) were isolated; they belong to polyacetylenes compounds [74].

Many plant crude extracts, however, have only been confirmed for their nematicidal activity without further identification and exploration of the specific active compounds. Ultrasound-assisted extraction with methanol was used to obtain the crude extracts of 14 *Asteraceae* plants. The extracts of *Eclipta prostrata* exhibited the highest activity upon the PWN (LC_50_/24 h = 0.36 μg/mL). The specific substance has not been identified [96]. Essential oils from 29 medicinal plant species were screened for nematicidal activity against the PWN. *Paeonia × suffruticosa*, *Perilla frutescens*, *Boswellia serrata*, and *Schizonepeta tenuifolia* essential oils demonstrated high efficacy, achieving 100% mortality in male, female, and juvenile nematodes at 2 mg/mL. The LC_50_ values against juveniles were 0.26, 0.41, 0.21, and 0.41 mg/mL, respectively [97]. The nematicidal activity of essential oils from 84 plant samples were evaluated. Among them, twenty essential oils demonstrated high mortality rates exceeding 96% at 2 μL/mL. *Ruta graveolens*, *Satureja montana*, and *Thymbra capitata* exhibited lethal concentrations (LC_100_) below 0.4 μL/mL. Fractionated components of these oils showed varying lethal concentrations, suggesting possible synergistic effects [98].

**Table 1:** Phytochemicals and some derivatives with potential for controlling *Bursaphelenchus xylophilus*.

Cat.	Species	Part	Compound Name	Extractant/ Solvent	Method	Nematicidal Activity	Ref.
Alkaloids	*Sophora alopecuroides*	Leaf	Aloperine	Methylbenzene	FF	LC50/5 d = 0.263 μg/mL	[33]
	*S. alopecuroides*	-	Aloperine	-	Injection	1 year, Control rate = 100%	[35]
	*S. alopecuroides*	-	Aloperine	-	FF	0.0001 g/mL, Reproductive inhibition rate = 100% (5 d)	[36]
	*S. alopecuroides*	-	Aloperine	Distilled water	DC	29.8%	[37]
	*S. alopecuroides*	-	Δ^11^-Dehydroaloperine	Distilled water	DC	5.8%	[37]
	*Peganum harmala*	Seed	Harmine	Ethanol	DC	LC50/48 h = 135.74 µg/mL	[43]
	*Piper peepuloides*	Fruit	Piperine	DMSO	FF	50 μg/mL, 100% (6 h)	[47]
	*Cephalotaxus fortunei*	Twig	Drupacine	Ethanol	DC	EC50/54 h = 27.1 µg/mL	[48]
	*Waltheria indica*	Root	5′-Methoxywaltherio-ne A	Methanol	DC	EC50/72 h = 2.13 μg/mL	[49]
	*W. indica*	Root	Waltherione A	Methanol	DC	EC50/72 h = 3.54 μg/mL	[49]
	*Clausena lansium*	Seed	Lansiumamide B	Methanol	DC	LC50/24 h = 8.38 mg/L	[50]
	*Orixa japonica*	Root	9-Methoxy-[1,3]dioxolo[4,5-b]-quinoline	Aqueous	DC	LC50/72 h = 12.66 μg/mL	[51]
	*O. japonica*	Root	Skimmianine	Aqueous	DC	LC50/72 h = 15.56 μg/mL	[51]
	*O. japonica*	Root	Kokusaginine	Aqueous	DC	LC50/72 h = 16.66 μg/mL	[51]
	*O. japonica*	Root	6-Acetonyldihyrochelerythrine	Aqueous	DC	LC50/72 h = 11.27 μg/mL	[51]
	*O. japonica*	Root, bark	(Z)-3-(4-Hydroxybenzylidene)-4-(4-hydroxyphenyl)-1-methylpyrrolidin-2-one	Ethanol	DC	LC50/72 h = 391.50 μg/mL	[74]
	*Heracleum hemsleyanum*	-	Reserpine	Ethanol	DC	LC50/72 h = 489.17 μM	[75]
	*Sophora flavescens*	Root	(–)-N-methylcytisine	Methanol	FF	log(1/ID50) = 7.91	[99]
	*S. flavescens*	Root	(–)-Anagyrine	Methanol	FF	log(1/ID50) = 7.92	[99]
	*S. flavescens*	Epigeal	Matrine	Methanol	FF	log(1/ID50) = 6.39	[100]
	*Macleaya cordata*	Stem, leaf	Sanguinarine	Ethanol	DC	LC50/24 h = 28.52 μg/mL	[101]
	*M. cordata*	Stem, leaf	Chelerytherine	Ethanol	DC	LC50/24 h = 34.50 μg/mL	[101]
	*M. cordata*	Stem, leaf	Allocryptopine	Ethanol	DC	LC50/24 h = 37.45 μg/mL	[101]
	*S. alopecuroides*	Seed	Sophoridine	Chloroform	DC	IC50/24 h = 0.822 μg/mL	[102]
	*S. alopecuroides*	Seed	Oxymatrine	Chloroform	DC	IC50/24 h = 0.722 μg/mL	[102]
	*S. alopecuroides*	Seed	Oxysophocarpine	Chloroform	DC	IC50/24 h = 0.622 μg/mL	[102]
Terpenes	-	-	Citronellol	Triton X-100	DC	LC50/4 h = 0.245 mg/mL (M) LC50/4 h = 0.235 mg/mL (F)	[56]
	-	-	Menthol	Triton X-100	DC	LC50/4 h = 0.985 mg/mL (M) LC50/4 h = 0.894 mg/mL (F)	[56]
	-	-	Nerol	Triton X-100	DC	LC50/4 h = 0.865 mg/mL (M) LC50/4 h = 0.926 mg/mL (F)	[56]
	-	-	Geraniol	Triton X-100	DC	LC50/4 h = 0.540 mg/mL (M) LC50/4 h = 0.415 mg/mL (F)	[56]
	-	-	Citral	Triton X-100	DC	LC50/4 h = 0.187 mg/mL (M) LC50/4 h = 0.139 mg/mL (F)	[56]
	-	-	Citronellal	Triton X-100	DC	LC50/4 h = 0.321 mg/mL (M) LC50/4 h = 0.298 mg/mL (F)	[56]
	-	-	Carvacrol	Triton X-100	DC	LC50/4 h = 0.125 mg/mL (M) LC50/4 h = 0.097 mg/mL (F)	[56]
	-	-	Thymol	Triton X-100	DC	LC50/4 h = 0.119 mg/mL (M) LC50/4 h = 0.110 mg/mL (F)	[56]
	*Thymus vulgaris*	-	Geraniol	Castor oil–ethanol	DC	LC50/24 h = 0.47 mg/mL	[57]
	*T. vulgaris*	-	Thymol	Castor oil–ethanol	DC	LC50/24 h = 1.08 mg/mL	[57]
	*T. vulgaris*	-	Carvacrol	Castor oil–ethanol	DC	LC50/24 h = 1.23 mg/mL	[57]
	*Trachyspermum ammi*, *Pimenta dioica*, *Litsea cubeba*	Seed, berry, fruit	Citral	Triton X-100	DC	LC50/24 h = 0.120 mg/mL	[58]
	*T. ammi*, *P. dioica*, *L. cubeba*	Seed, berry, fruit	Neral	Triton X-100	DC	LC50/24 h = 0.525 mg/mL	[58]
	*Michelia gioi*	Leaf	Parthenolide	Ethyl acetate	DC	200 μg/mL, 70.4% (48 h)	[59]
	*Dictamnus dasycarpus*	Bark	Evodin	Ethyl acetate	DC	LC50/72 h = 17.91 μg/mL	[60]
	*D. dasycarpus*	Bark	Obacunone	Ethyl acetate	DC	LC50/72 h = 15.99 μg/mL	[60]
	*D. dasycarpus*	Bark	Fraxinellone	Ethyl acetate	DC	LC50/72 h = 9.78 μg/mL	[60]
	*Euphorbia kansui*	Root	3-O-(2″, 3″-Dimethylbutanoyl)-13-O-dodecanoylingenol	Ethanol	FF	5 μg, Antinematodal activity	[61]
	*E. kansui*	Root	3-O-(2″, 3″-Dimethylbutanoyl)-13-O-decanoylingenol	Ethanol	FF	5 μg, Antinematodal activity	[61]
	*Magnolia grandiflora*	Branch, leaf	4,5-Epoxy-1(10)E,11(13)-germacradien-12,6-olide	Ethyl acetate	DC	LC50/72 h = 71 µg/mL	[62]
Phenylpropanoids	*T. ammi*, *P. dioica*, *L. cubeba*	Seed, berry, fruit	Methyl isoeugenol	Triton X-100	DC	LC50/24 h = 0.21 mg/mL	[58]
	*T. ammi*, *P. dioica*, *L. cubeba*	Seed, berry, fruit	Isoeugenol	Triton X-100	DC	LC50/24 h = 0.20 mg/mL	[58]
	*T. ammi*, *P. dioica*, *L. cubeba*	Seed, berry, fruit	Eugenol	Triton X-100	DC	LC50/24 h = 0.48 mg/mL	[58]
	*T. ammi*, *P. dioica*, *L. cubeba*	Seed, berry, fruit	Methyl eugenol	Triton X-100	DC	LC50/24 h = 0.517 mg/mL	[58]
	*Valeriana jatamansi*	Root	Cis-asarone	Triton X-100	DC	1 mg/mL, 100% (24 h)	[63]
	*Liquidambar orientalis*	Resin	Trans-cinnamyl alcohol	Triton X-100	DC	1 mg/mL, 100% (24 h)	[63]
	-	-	(E)-cinnamaldehyde	Castor oil–ethanol	DC	LD50/24 h = 0.057 mg/mL	[64]
	-	-	α-Methyl-(E)-cinnamaldehyde	Castor oil–ethanol	DC	LD50/24 h = 0.131 mg/mL	[64]
	-	-	(E)-4-methoxycinnamaldehyde	Castor oil–ethanol	DC	LD50/24 h = 0.262 mg/mL	[64]
	-	-	(E)-2-methoxycinnamaldehyde	Castor oil–ethanol	DC	LD50/24 h = 0.270 mg/mL	[64]
	-	-	Ethyl cinnamate	Castor oil–ethanol	DC	LC50/24 h = 0.114 mg/mL	[64]
	-	-	Methyl cinnamate	Castor oil–ethanol	DC	LC50/24 h = 0.163 mg/mL	[64]
	-	-	Allyl cinnamate	Castor oil–ethanol	DC	LC50/24 h = 0.195 mg/mL	[64]
	-	-	Ethyl α-cyanocinnamate	Castor oil–ethanol	DC	LC50/24 h = 0.333 mg/mL	[64]
	*Cinnamomum verum*	Bark	Cinnamyl acetate	Triton X-100	DC	LC50/4 h = 32.81 µL/L	[65]
	*Kaempferia galanga*	-	Ethyl *ρ*-methoxy cinnamate	Methanol	DC	LC50/72 h = 2.81 mg/L	[66]
	*K. galanga*	-	Ethyl cinnamate	Methanol	DC	LC50/72 h = 29.7 mg/L	[66]
	*Zanthoxylum armatum*	Fruit	Methyl trans-cinnamate	Distilled water (Triton X-100)	DC	2.0 mg/mL, 100%	[67]
	*Z. armatum*	Fruit	Ethyl trans-cinnamate	Distilled water (Triton X-100)	DC	2.0 mg/mL, 100%	[67]
	*K. galanga*	Root	Ethyl trans-cinnamate	Methanol	DC	60 μg/mL, 100% (4 h)	[68]
	*K. galanga*	Root	Ethyl *ρ*-methoxycinnamate	Methanol	DC	60 μg/mL, 100% (4 h)	[68]
	*Zostera marina*	-	Rosmarinic acid	Ethanol	DC	LC50/24 h = 1.18 mg/g	[69]
Coumarins	*Heracleum candicans*	Root	8-Geranyloxypsoralen	Ethanol	DC	LC50/72 h = 117.5 mg/L	[71]
	*H. candicans*	Root	Imperatorin	Ethanol	DC	LC50/72 h = 179.0 mg/L	[71]
	*H. candicans*	Root	Heraclenin	Ethanol	DC	LC50/72 h = 148.7 mg/L	[71]
	*Stellera chamaejasme*	Root	Umbelliferone	Ethanol	DC	LC50/72 h = 3.3 μM	[72]
	*S. chamaejasme*	Root	Daphnoretin	Ethanol	DC	LC50/72 h = 65.3 μM	[72]
	*Ficus carica*	Leaf	Psoralen	Ethanol	DC	LC50/72 h = 115.03 μg/mL	[73]
	*Hansenia weberbaueriana*	Root	Columbianetin	Ethanol	DC	LC50/72 h = 21.83–103.44 µg/mL	[74]
	*H. weberbaueriana*	Root	Isoimperatorin	Ethanol	DC	LC50/72 h = 17.21–30.91 µg/mL	[74]
	*H. hemsleyanum*	-	Columbianadin	Ethyl acetate	DC	LC50/72 h = 406.74 μM	[75]
	*F. carica*	Leaf	Psoralen	Ethanol	DC	LC50/72 h = 463.32 μM	[75]
	*F. carica*	Leaf	Bergapten	Ethanol	DC	LC50/72 h = 430.08 μM	[75]
	*Angelica pubescens*	Root	Osthole	Ethyl acetate	DC	LC50/72 h = 489.17 μM	[76]
	*A. pubescens*	Root	Xanthotoxin	Ethyl acetate	DC	LC50/72 h = 435.66 μM	[76]
	*Cnidium monnieri*, *Angelica dahurica*	Fruit (C), root (A)	Osthole	Ethanol	DC	LC50/72 h = 64.93 µg/mL	[77]
	*C. monnieri*, *A. dahurica*	Fruit (C), root (A)	Xanthotoxin	Ethanol	DC	LC50/72 h = 54.68 µg/mL	[77]
	*C. monnieri*, *A. dahurica*	Fruit (C), root (A)	Cindimine	Ethanol	DC	LC50/72 h = 24.73 µg/mL	[77]
	*C. monnieri*, *A. dahurica*	Fruit (C), root (A)	Isopimpinellin	Ethanol	DC	LC50/72 h = 92.16 µg/mL	[77]
	*C. monnieri*, *A. dahurica*	Fruit (C), root (A)	Marmesin	Ethanol	DC	LC50/72 h = 122.96 µg/mL	[77]
	*C. monnieri*, *A. dahurica*	Fruit (C), root (A)	Isoimperatorin	Ethanol	DC	LC50/72 h = 43.08 µg/mL	[77]
	*C. monnieri*, *A. dahurica*	Fruit (C), root (A)	Imperatorin	Ethanol	DC	LC50/72 h = 35.72 µg/mL	[77]
	*C. monnieri*, *A. dahurica*	Fruit (C), root (A)	Bergapten	Ethanol	DC	LC50/72 h = 52.07 µg/mL	[77]
Flavonoids	*S. chamaejasme*	Root	(+)-Chamaejasmine	Ethanol	DC	LC50/72 h = 4.7 μM	[72]
	*S. chamaejasme*	Root	Ruixianglangdusu B	Ethanol	DC	LC50/72 h = 15.7 μM	[72]
	*S. chamaejasme*	Root	Chamaejasmenin C	Ethanol	DC	LC50/72 h = 2.7 μM	[72]
	*S. chamaejasme*	Root	7-Methoxyneochamaejasmin A	Ethanol	DC	LC50/72 h = 167.3 μM	[72]
	*S. chamaejasme*	Root	Chamaechromone	Ethanol	DC	LC50/72 h = 36.7 μM	[72]
	*S. chamaejasme*	Root	Isosikokianin A	Ethanol	DC	LC50/72 h = 2200 μM	[72]
	-	-	Rotenone	Acetone	DC	LC50/120 h = 1.86 μg/mL	[79]
	-	-	2-Phenyl chromone	DMSO	FF	LC50/24 h = 100 μM	[83]
Polyphenols	*Punica granatum*	Rind	Punicalin	Aqueous	DC	LC50/72 h = 826.96 μM	[75]
	*P. granatum*	Rind	Corilagin	Aqueous	DC	LC50/72 h = 868.28 μM	[75]
	*P. granatum*	Bark	Punicalagin	Aqueous	DC	LC50/72 h = 307.08 μM	[75]
Naphthoquinones	-	-	1,4-Naphthoquinones	DMSO	DC	LC50/48 h = 100 ppm	[103]
	-	-	Juglone	DMSO	DC	LC50/48 h = 57 ppm	[103]
	-	-	Plumbagin	DMSO	DC	LC50/48 h = 104 ppm	[103]
Polyacetylenes	*H. weberbaueriana*	Root	Falcarinol	Ethanol	DC	LC50/72 h = 7.42 µg/mL	[74]
	*H. weberbaueriana*	Root	Falcarindiol	Ethanol	DC	LC50/72 h = 0.95 µg/mL	[74]
Glycosides	*Liriope muscari*	Root	1,4-Epoxy-cis-eudesm-6-O-β-D-glucopyranoside	Ethanol	DC	LC50/72 h = 339.76 μg/mL	[88]
	*L. muscari*	Root	1β,6β-Dihydroxy-cis-eudesm-3-ene-6-O-β-D-glucopyranoside	Ethanol	DC	LC50/72 h = 82.84 μg/mL	[88]
	*L. muscari*	Root	1α,6β-Dihydroxy-cis-eudesm-3-ene-6-O-β-D-glucopyranoside	Ethanol	DC	LC50/72 h = 153.39 μg/mL	[88]
	*L. muscari*	Root	1α, 6β-dihydroxy-5, 10-bis-epi-eudesm-4(15)-ene-6-O-β-D-glucopyranoside	Ethanol	DC	LC50/72 h = 465.68 μg/mL	[88]
Sulfides	*Allium sativum*	Bulb	Diallyl disulphide	Triton X-100	DC	LC50/4 h = 37.06 µL/L	[65]
	*A. sativum*	Bulb	Diallyl trisulphide	Triton X-100	DC	LC50/4 h = 2.79 µL/L	[65]
	*Allium cepa*	-	Dipropyl trisulfide	Ethanol	DC	LC50/24 h = 5.01 μg/mL	[90]
	*A. cepa*	-	Methyl propyl trisulfide	Ethanol	DC	LC50/24 h = 16.60 μg/mL	[90]
	-	-	Allyl isothiocyanate	Distilled water	DC	LC50/24 h = 0.000271%	[91]
	*A. cepa*	-	Propyl trisulphide	Triton X-100	DC	LC50/24 h = 5 µg/mL	[104]
	*A. cepa*	-	Methyl propyl trisulphide	Triton X-100	DC	LC50/24 h = 22.9 µg/mL	[104]
Aliphatic compounds	*Coriandrum sativum*	Herb	Trans-2-decenal	Triton X-100	DC	1 mg/mL, 100% (24 h)	[63]
	*C. sativum*	Herb	Octanal	Triton X-100	DC	1 mg/mL, 89.0% (24 h)	[63]
	*C. sativum*	Herb	Nonanal	Triton X-100	DC	1 mg/mL, 95.8% (24 h)	[63]
	*C. sativum*	Herb	Decanal	Triton X-100	DC	1 mg/mL, 100% (24 h)	[63]
	*C. sativum*	Herb	Undecanal	Triton X-100	DC	1 mg/mL, 98.7% (24 h)	[63]
	*C. sativum*	Herb	Dodecanal	Triton X-100	DC	1 mg/mL, 86.3% (24 h)	[63]
	*C. sativum*	Herb	Trans-2-decen-1-ol	Triton X-100	DC	0.2 mg/mL, 98% (24 h)	[63]
	*C. sativum*	Herb	Decanol	Triton X-100	DC	0.2 mg/mL, 100% (24 h)	[63]
	-	-	3-Methylbutyl tiglate	Ethanol	DC	LC50/48 h = 0.0218 mg/mL	[92]
	-	-	Isobutyl 2-methylbutanoate	Ethanol	DC	LC50/48 h = 0.0284 mg/mL	[92]
	-	-	3-Methylbutyl 2-methylbutanoate	Ethanol	DC	LC50/48 h = 0.0326 mg/mL	[92]
	-	-	3-Methyl-2-butenyl 2-methylbutanoate	Ethanol	DC	LC50/48 h = 0.0402 mg/mL	[92]
	-	-	Pentyl 2-methylbutanoate	Ethanol	DC	LC50/48 h = 0.0480 mg/mL	[92]
	-	-	Trans-2-hexenal	Distilled water	DC	LC10/48 h = 0.162 μL/L	[93]
Thiophenes	*O. japonica*	Root	α-Terthienyl	Aqueous	DC	LC50/72 h = 1.95 μg/mL	[51]
	*Eclipta prostrata*	-	Terthiophene	Methanol	DC	1.00 ppm, 92.8% (24 h)	[105]
Aromatic compounds	*L. orientalis*	Resin	Benzaldehyde	Triton X-100	DC	1 mg/mL, 94.1% (24 h)	[63]
	*Z. armatum*	Fruit	Methyl salicylate	Distilled water (Triton X-100)	DC	2.0 mg/mL, 100%	[67]
	*Z. armatum*	Fruit	Ethyl salicylate	Distilled water (Triton X-100)	DC	2.0 mg/mL, 100%	[67]
Lactones	*Conioselinum tenuissimum*	Root	(Z)-Ligustilide	Ethanol	DC	LC50/24 h = 0.24 mg/mL	[95]
Camptothecin derivatives	-	-	7-CH_2_C_6_H_5_-camptothecin (CPT)	Acetone	DC	LC_50_/24 h = 2.28 mg/L	[38]
	-	-	7-CHO-CPT	Acetone	DC	LC_50_/24 h = 2.21 mg/L	[38]
	-	-	7-CH_2_OC-OC_6_H_5_-CPT	Acetone	DC	LC_50_/24 h = 1.37 mg/L	[38]
	-	-	10-CH_2_OCOC_6_H_5_-CPT	Acetone	DC	LC_50_/24 h = 1.68 mg/L	[38]
	-	-	20-CH_2_OCOC_6_H_5_-CPT	Acetone	DC	LC_50_/24 h = 0.31 mg/L	[38]
	-	-	20-F-CPT	Acetone	DC	LC_50_/24 h = 1.71 mg/L	[38]
	-	-	20-(S)-CPT	Acetone	DC	LC50/24 h = 12.18 mg/L	[38]
	-	-	7-(1-(4-methoxybenzoyl)piperazin-4-yl)-methyl-camptothecin	Acetone	DC	LC50/24 h = 6.34 mg/L	[39]
	-	-	7-(1-(2-methoxybenzoyl)piperazin-4-yl)methyl-camptothecin	Acetone	DC	LC50/24 h = 6.53 mg/L	[39]
	-	-	N-(2,4,6-trimethoxybenzyl)-β-carboline-3-carbohydrazide	Acetone	DC	LC50/24 h = 42.49 μg/mL	[44]
Harmine derivatives	-	-	Harmine quaternary ammonium derivatives 10	Distilled water (2% DMSO)	DC	LC50/48 h = 1.63 μg/mL	[46]
	-	-	Harmine quaternary ammonium derivatives 11	Distilled water (2% DMSO)	DC	LC50/48 h = 1.63 μg/mL	[46]
	-	-	Harmine quaternary ammonium derivatives 12	Distilled water (2% DMSO)	DC	LC50/48 h = 1.75 μg/mL	[46]
	-	-	Harmine quaternary ammonium derivatives 13	Distilled water (2% DMSO)	DC	LC50/48 h = 1.44 μg/mL	[46]
Nitrile derivatives	-	-	4-Methoxycinnamonitrile	Castor oil–ethanol	DC	LC50/24 h = 0.224 mg/mL	[64]
	-	-	Cinnamonitrile	Castor oil–ethanol	DC	LC50/24 h = 0.448 mg/mL	[64]
	-	-	Cinnamyl bromide	Castor oil–ethanol	DC	LC50/24 h = 0.502 mg/mL	[64]
Aliphatic derivatives	-	-	C_9_-C_11_ alkanols, C_10_-C_11_ 2E-alkenols, C_8_-C_9_ 2E-alkenals, C_9_-C_10_ alkanoic acids	Triton X-100	DC	0.125 mg/mL, >80% (48 h)	[94]
Matrine derivatives	-	-	Sophocarpine	Methanol	FF	log(1/ID50) = 7.78	[100]
	-	-	Sophoramine	Methanol	FF	log(1/ID50) = 6.68	[100]
	-	-	Cytisine	Methanol	FF	log(1/ID50) = 8.23	[100]
	-	-	N-Nethylcytisine	Methanol	FF	log(1/ID50) = 7.91	[100]
	-	-	Anagyrine	Methanol	FF	log(1/ID50) = 7.54	[100]
	-	-	Sparteine	Methanol	FF	log(1/ID50) = 7.96	[100]
3-Acylbarbituric acid analogues	-	-	3-Acylbarbituric acid analogues-18	Ethanol	DC	10 µg/mL, 93.4%	[106]
Sulfonate derivatives	-	-	Sulfonate derivatives of maltol 3M	Acetone	DC	LC50/24 h = 1.1762 mg/L	[107]
	-	-	Sulfonate derivatives of maltol 3N	Acetone	DC	LC50/24 h = 1.2384 mg/L	[107]
Extracts	*P. harmala*	Seed	Fraction A_6_	Ethanol	DC	LC50/24 h = 86.02 μg/mL	[43]
	*E. prostrata*	Whole plant	-	Methanol	DC	LC50/24 h = 0.36 μg/mL	[96]
	*Paeonia × suffruticosa*	Root	-	Triton X-100	DC	LC50/4 h = 0.26 mg/mL	[97]
	*Perilla frutescens*	Leaf	-	Triton X-100	DC	LC50/4 h = 0.41 mg/mL	[97]
	*Boswellia serrata*	Resin	-	Triton X-100	DC	LC50/4 h = 0.21 mg/mL	[97]
	*Schizonepeta tenuifolia*	Whole plant	-	Triton X-100	DC	LC50/4 h = 0.41 mg/mL	[97]
	*Thymbra capitata*	Aerial part	-	Aqueous	DC	LC100/24 h = 0.375 μL/mL	[98]
	*Satureja montana*	Aerial part	-	Aqueous	DC	LC100/24 h = 0.374 μL/mL	[98]
	*Ruta graveolens*	Aerial part	-	Aqueous	DC	LC100/24 h = 0.358 μL/mL	[98]
	*Origanum vulgare*	Aerial part	-	Aqueous	DC	LC100/24 h = 1.606 μL/mL	[98]
	*Cymbopogon citratus*	Leaf	-	Aqueous	DC	LC100/24 h = 1.059 μL/mL	[98]
	*Pinellia ternata*	Tuber	Total alkaloids	Chloroform	DC	IC50/24 h = 16.18 μg/mL	[102]
	*S. alopecuroides*	Seed	Total alkaloids	Chloroform	DC	IC50/24 h = 0.622 μg/mL	[102]
	*Artemisia capillaris*	Leaf, stem	-	Methanol	FF	20 mg, Propagation Rate = 0.1%	[108]
	*Cirsium japonicum*	Root	-	Methanol	FF	20 mg, Propagation Rate = 0.1%	[108]
	*Coreopsis lanceolata*	Flower	-	Methanol	FF	20 mg, Propagation Rate = 0.2%	[108]
	*Erigeron annuus*	Root	-	Methanol	FF	20 mg, Propagation Rate = 0.1%	[108]
	*Sauropus androgynus*	Shoot	-	Methanol	FF	0.625 mg/cotton ball, Minimum effective dose	[109]
	*Eugenia polyantha*	Leaf	-	Methanol	FF	0.625 mg/cotton ball, Minimum effective dose	[109]
	*Areca catechu*	Fruit	-	Methanol	FF	0.625 mg/cotton ball, Minimum effective dose	[109]
	*Piper betle*	Leaf	-	Methanol	FF	0.625 mg/cotton ball, Minimum effective dose	[109]
	*Piper nigrum*	Berry	-	Methanol	FF	0.625 mg/cotton ball, Minimum effective dose	[109]
	*Bischofia javanica*	Sap	-	Methanol	FF	0.7 mg/cotton ball, Minimum effective dose	[110]
	*A. catechu*	Seed	-	Methanol	FF	0.7 mg/cotton ball, Minimum effective dose	[110]
	*Knema hookeriana*	Sap	-	Methanol	FF	0.7 mg/cotton ball, Minimum effective dose	[110]
	*Melia azedarach*	Bark, fruit	-	Ethanol	DC	100 mg/kg, 96%	[111]
	*Paraderris elliptica*	Root	-	Acetone	FF	Proliferation rate = 0	[112]
	*Nerium oleander*	Leaf	-	Ethanol	DC	1.2 mg/mL, 97.1%	[113]
	*Tetraena mongolica*	Stem, leaf	-	Aqueous	DC	LC50/24 h = 1.18 mg/mL	[114]
	*Camellia sinensis*	Seed	-	Ethanol	DC	LC50/60 h = 0.0119 mg/mL	[115]
	*C. sinensis*	-	-	Ethanol	DC	LC50/60 h = 0.5145 mg/mL	[116]
	*M. azedarach*	-	-	Ethanol	DC	LC50/60 h = 0.6100 mg/mL	[116]
	*Pterocarya stenoptera*	-	-	Ethanol	DC	LC50/60 h = 0.8064 mg/mL	[116]
	*T. mongolica*	-	-	Methanol	DC	41.25 mg/mL, 92.36% (8 h)	[117]
	*Yulania cylindrica*	-	-	Aqueous	DC	LC50/24 h = 947.10 μL/L	[118]
	*Torreya grandis*	-	-	Aqueous	DC	LC50/24 h = 960.47 μL/L	[118]
	*Helianthemum ordosicum*	-	-	Aqueous	DC	LC50/5 d = 1.21 g/mL	[119]
	*Ammopiptanthus mongolicus*	Stem, leaf	-	Aqueous	DC	LC50/24 h = 0.56 g/L	[120]
	*P. harmala*	Stem, leaf	-	Aqueous	DC	LC50/24 h = 1.39 g/L	[120]
	*Vincetoxicum mongolicum*	-	-	Methanol	DC	2 g/L, 100% (48 h)	[121]
	*S. montana*	Leaf	-	Aqueous	DC	LC100/24 h = 0.858 mg/mL	[122]
	*T. capitata*	Flower	-	Aqueous	DC	LC100/24 h = 0.985 mg/mL	[122]
	*Thymus caespititius*	Flower	-	Aqueous	DC	LC100/24 h, 0.874 mg/mL	[122]
	*Tagetes erecta*	Root	-	Ethanol	DC	LC50/72 h = 6.3 mg/L	[123]
	*Camellia oleifera*	Camellia cake	-	Aqueous	DC	10 mg/mL, 100% (48 h)	[124]
	*Tripterygium wilfordii*	Root	-	Aqueous	DC	10 mg/mL, 88.9% (48 h)	[124]
	*R. graveolens*, *S. montana*, *T. capitata*	-	-	Distilled water	DC	LC100/24 h < 0.4 µL/mL	[125]
	*Foeniculum vulgare*	-	-	Ethanol	DC	LC50/24 h = 16.5 mg/mL	[126]
	*Chromolaena odorata*	Aerial part	-	Ethanol	DC	LC50/72 h = 0.6892 g/L	[127]
	*Ageratina adenophora*	Aerial part	-	Ethanol	DC	LC50/72 h = 0.6813 g/L	[127]
	*Mikania micrantha*	Aerial part	-	Ethanol	DC	LC50/72 h = 0.7474 g/L	[127]
	*Rheum palmatum*	Root	-	Aqueous	DC	LC50/72 h = 0.067 g/L	[128]
	*M. cordata*	Leaf	-	Aqueous	DC	20 mg/mL, 73.3% (72 h)	[129]
	*Ginkgo biloba*	Episperm	-	Aqueous	DC	20 mg/mL, 84.9% (72 h)	[130]
	*Zanthoxylum schinifolium*	Peel	-	Aqueous	DC	LC50/4 h = 0.625 mg/mL	[131]
	*Bidens pilosa*	-	-	Aqueous	DC	10 percent solution, 100% (7–8 d)	[132]
	*B. pilosa*	-	-	Aqueous	DC	10 percent solution, 100% (7–8 d)	[132]
	*Alternanthera philoxeroides*	Whole plant	-	Ethyl acetate	DC	LC50/72 h = 0.5888 g/L	[133]
	*Causonis japonica*	Whole plant	-	Aqueous	DC	LC50/72 h = 0.9693 g/L	[133]
	*Chamaecyparis pisifera*	Stem, leaf	-	Aqueous	DC	LC50/72 h = 2.84 mg/mL	[134]
	*Chamaecyparis obtusa*	Stem, leaf	-	Aqueous	DC	LC50/72 h = 1.76 mg/mL	[134]
	*Backhousia citriodora*	Stem, leaf	-	Distilled water	DC	LC50/72 h = 85.56 μg/mL	[135]

Note: Cat.: chemical category; Ref.: reference; M: male; F: female; d: day; C: *Cnidium monnieri*; A: *Angelica dahurica*; DC: direct contact bioassay; FF: fungal-feeding assay.

## 5. Influence Factors on the Nematicidal Activity of Phytochemicals

PCs exhibit great potential in the control of the PWN. However, their commercialization and production are limited by the lack of comprehensive research. Nematicidal activity is one of the most important indicators for assessing the potential application prospects, it will be influenced by many factors. Therefore, it is crucial to thoroughly understand these influencing factors on the nematicidal activity of PCs.

PCs, including plant extracts, plant essential oils, and plant secondary metabolites, are single compounds or mixtures. They may interact antagonistically or synergistically among components. All kinds of influencing factors, such as the extraction process, compound characteristics, environmental conditions, genetic factors, experimental experiences, and nematode population can influence their nematicidal activity. Extraction processes can impact the efficacy of active components. For example, the ethanol extract of *Paeonia × suffruticosa* exhibited a smaller LC_50_/24 h (0.306 g/L) than ethyl acetate (1.593 g/L) [128]. The lower concentration of *Bidens pilosa* aqueous extract showed a lower nematicidal activity [132]. Different solvents can lead to varying amounts of dissolution, which finally affect the nematicidal activity. In terms of composition, when the active components within the mixture possess synergistic effects or the proportion of highly nematicidal components is higher, the nematicidal activity will be enhanced. Research has shown that the combined hydrocarbon and oxygen-containing fractions of monoterpene-rich essential oils showed effective nematicidal activity through additive and/or synergistic relations [125]. The composition of essential oils depend greatly on the plant genotype and genetic factors, plant parts and anatomical organs, environmental and soil conditions, developmental stage, stress conditions, and other factors [18].

From the perspective of compound structure, the type and position of functional groups are the main factors affecting nematicidal activity, as well as the position, quantity, length, saturation, and configuration of the chemical bonds [58,67,94,128]. In general, compounds with phenol, alcohol, or aldehyde functional groups showed the highest activities, while hydrocarbons or ketones were less effective against the PWN [18]. Oxygen-containing aliphatic compounds exhibited different levels of nematicidal activity, as C_12_H_26_O > C_13_H_28_O > C_11_H_24_O > C_10_H_22_O [136]. Trans-cinnamate showed good nematicidal activity, and its activity was related to the presence of a carbonyl group and a double bond at the α,β-position of the carbonyl group [67]. A functional group at the C1 position in the benzene ring was particularly important for the nematicidal activity of eugenol [58]. Some specific findings suggested that thymol and α-terpineol exhibited surprisingly higher anti-PWN activity after glycosylation [137].

Different environmental factors, such as temperature, humidity, and light directly affect the volatility, degradation rate, and insecticidal efficacy of PCs. The LC_50_/24 h of the methanol extract of *Eclipta prostrata* was 0.36 μg/mL with a phototoxicity ratio of 7.19 under light exposure [96]. The response time and efficacy also depend on the gender and age of the nematodes [56]. The accuracy of nematode counting under a microscope mainly relies on the observers’ experience, leading into subjective factors that affect the results. Moreover, isolates of the PWN from different regions and populations will also exhibit variations in their response to the same substance [18].

## 6. Research Progress of the Nematicidal Mechanism of Phytochemicals

Investigation into the mechanism of PCs can provide theoretical support for the development of new nemacides, which might be beneficial in reducing reliance on chemical nemacides, and minimize the negative impacts on the environment. Currently, research concerning the mechanism of PCs on anti-PWN lack systematic and in-depth investigation. Only about 12% of the total documents refer to the nematicidal mechanism. However, the key factors leading to nematode death have not yet been determined for most compounds. Recent studies mainly focus on detoxification enzyme systems, including the inhibitory effects on the activity of CAT and GST (glutathione S-transferase). Some research includes the study of neurotransmitters participating in neural excitation conduction, such as the changes in acetylcholine content and ACE activity. Additionally, there is also some research focusing on the changes in total sugar and protein content, as well as the damage to the body wall and digestive tract [45]. For instance, inhibition activity of ACE and GST by 63 aliphatic compounds was evaluated, C_12_ 2E-alkenal had an IC_50_ of 0.0059 mg/mL on BxACE-2. C_6_, C_9_, and C_10_ 2E-alkenals and C_12_ alkanoic acid all showed more than 45% GST inhibition activity [138]. Trans-2-hexenal inhibited the dispersal of the PWN, affected nutrient metabolism and digestive enzyme activity, and increased the GST activity [93]. According to the enzyme assays in vitro, punicalagin could inhibit the activity of ACE (IC_50_ = 0.60 mM), amylase (0.96 mM), and cellulase (1.24 mM) [87]. The derivative of harmaline and harmine exhibited inhibitory activity against ACE [45,46].

PCs can also indirectly control the PWN by enhancing the resistance of pine trees. For example, after treating with methyl jasmonate, salicylic acid, and benzo (1, 2, 3)-thiadiazole-7-carbothioic acid-S-methyl ester, the resistance in *Pinus pinaster* to the PWN was enhanced by boosting the antioxidant system, altering the accumulation of micronutrients, and influencing the diversity of bacteria associated with the plants [139].

## 7. Conclusions and Future Prospects

PCs exhibit many special features for potential application, including nontarget and environmentally friendly activity, high efficacy in nematode control, easy degradation, and rich resources. Research on the application of PCs for the control of PWD is increasing, which is accompanied by more effective active substances that have been identified and more advanced technologies that have been applied. The main challenges currently facing the application of PCs include the lack of standardized extraction and formulation techniques, poor standardization and quality control of the required active ingredients, and the absence of reasonable regulatory procedures and pricing [140]. Based on the current demand and potential development, some prospects are suggested to help expand the practical application of PCs on PWD management.

(1) Comprehensive understanding of PC characteristics is the prerequisite for commercialization and production. Although PCs have drawn increasing interest on the control of PWD caused by the PWN, there are only a few commercial productions and pesticides. By 2023, over a thousand substances and derivatives were proved to exhibit nematicidal activity against the PWN, and more than a hundred have shown significant activity. However, only *Sophora flavescens* extract, which contains 1% matrine, has been registered as a pesticide for the PWN in China. Many studies assess nematicidal activity in the laboratory and only some simple characteristics are summarized, lacking in-depth research on actual outdoor effects, PC characteristics, and mechanisms of action. For example, some indoor active PCs are hydrophobic and easily degraded in the environment [81]. Therefore, despite ongoing research on the discovery of more plants and active substances, the in-depth comprehensive studies on some excellent ingredients would actually accelerate the procedure for commercializing and producing PC pesticides.
(2) Improving the effectiveness of PCs against the PWN would be the continuous topics and difficulties. It is necessary to optimize the extraction process, which could provide more effective compounds with higher concentrations. The structure–activity relationship among substances is also an important reason for differences in nematicidal effectiveness. Utilizing organic chemistry to understand and modify the structure can potentially enhance the nematicidal effect of substances [18]. The development of nanotechnology in recent years has provided new approaches to improve the control effectiveness. For example, through efficient loading or encapsulation of active ingredients using nanocarriers, the water solubility, stability, and sustained release of substances can be enhanced [141]. Moreover, synergistic enhancement through the combination of PCs and chemical pesticides might be a simple, effective, and low-cost improvement method in the control of the PWN [142], which requires efforts to screen the suitable compounds, ratios, and additives.
(3) Clarifying the potential PC mechanisms of action would facilitate their further application against the PWN. Most research is relatively limited as yet, mainly involving the effects on enzyme activities, differentially expressed genes, and detoxification regulatory pathways. Although some explorations provide a wealth of theoretical data, these results are insufficient to reveal the complex biological processes. To understand the mechanisms, it is necessary to focus on the target proteins, and the potential aspects on the interaction between active PCs with targets, including target identification and interaction, signal induction and conduction, gene expression and regulation, chemical functional group and activity, synergistic compound and effects, etc. With further development in molecular biology techniques and interdisciplinary intersection, i.e., cytotoxicology, bioinformatics, microimaging, synthetic biology, structural chemistry, nanomaterials science, artificial intelligence, and some others, it will be more efficient and more accurate to explore potential mechanisms.

Overall, this review summarizes and analyzes the application of PCs in the control of PWD. Most aspects have been covered, such as the identification of effective substances with high application potential, the analysis of extraction and identification processes for active ingredients, and the investigation of nematicidal activity, influencing factors, mechanisms of action, and research status. All of these factors provide a theoretical basis for the application of PCs.

## Supplementary Materials

The following supporting information can be downloaded at: https://www.mdpi.com/article/10.3390/plants13152129/s1, Table S1: Original literatures and data of plants, phytochemicals and derivatives used for controlling pine wilt disease; Table S2: Classification and statistics of plants, phytochemicals and derivatives used for controlling pine wilt disease; Table S3: Proportion of different fungi culturing pine wood nematodes; Table S4: Calculation of nematicidal activity method proportion indoors.
